# Explicit motor learning interventions are still relevant for ACL injury rehabilitation: do not put all your eggs in the implicit basket!

**DOI:** 10.1136/bjsports-2020-103643

**Published:** 2021-02-15

**Authors:** Elmar Kal, Toby Ellmers, Jed Diekfuss, Marinus Winters, John van der Kamp

**Affiliations:** 1 College of Health, Medicine and Life Sciences; Department of Health Sciences, Brunel University London, London, UK; 2 College of Health, Medicine and Life Sciences; Centre for Cognitive Neuroscience, Brunel University London, London, UK; 3 School of Sport and Health Sciences, University of Exeter, Exeter, UK; 4 The SPORT Center, Division of Sports Medicine, Cincinnati Children's Hospital Medical Center, Cincinnati, Ohio, USA; 5 Department of Orthopaedics, Emory University School of Medicine, Atlanta, Georgia, USA; 6 Research Unit for General Practice in Aalborg, Department of Clinical Medicine, Aalborg University Faculty of Health Sciences, Aalborg, Denmark; 7 Department of Human Movement Sciences, Faculty of Behavioural and Movement Sciences, Vrije Universiteit Amsterdam, Amsterdam, The Netherlands; 8 Research Centre for Exercise, School and Sport, Windesheim University of Applied Sciences, Zwolle, The Netherlands

**Keywords:** sports rehabilitation programmes, knee injuries, ACL

## Introduction

ACL ruptures are rapidly increasing.^1^ ACL injuries can have a profound impact on an athlete’s physical and psychological functioning, and sporting career. Current standard of care following ACL injury is neuromuscular rehabilitation to help athletes regain motor skills. Optimising *how* rehabilitation is delivered has the potential to further enhance motor relearning and reduce the risk of secondary knee injuries.^2^

ACL injury rehabilitation programmes aiming to reduce secondary injury risk often involve *explicit learning* strategies to improve biomechanics and increase neuromuscular control.^3^ Athletes are mostly instructed to consciously control movements using internally focused, verbal cues that prescribe desired movement patterns (eg, ‘do not bring your knees over your toes’ during squatting). Only with sustained practice does explicit learning result in consistent, fluent, and automatic, motor performance.

Recently, practitioners have been encouraged to minimise explicit learning during ACL injury rehabilitation, and use *implicit learning* interventions such as external focus cues.^2^ We contend that implicit interventions are not a panacea, and that explicit interventions remain important in ACL injury rehabilitation. We discuss key individual characteristics and contextual constraints that warrant the use of explicit interventions.

### Why would implicit learning be beneficial to ACL rehabilitation?

The *theoretical* advantage of implicit learning interventions is that athletes reach automaticity earlier in the learning process. Accordingly, implicit learning should reduce the risk of reinjury as:

- Athletes will have more attentional resources available to deal with high cognitive–perceptual demands of events that often precipitate ACL injuries (eg, an approaching defender or incoming ball).
- Athletes will be less likely to fall back on using verbal rules to consciously control movement, especially when anxious or fatigued. This enables them to ‘self-organise’ and flexibly adapt to quickly changing task demands (eg, a rapid change in direction of movement).

### Evidence for implicit learning in the context of ACL injury

There is evidence that interventions that aim to induce implicit learning can improve preliminary outcomes *associated* with ACL injury risk (eg, knee biomechanics in standardised laboratory tasks).^4^ However, changes in biomechanics may not readily translate to actual changes in injury risk. It is also unclear whether improved biomechanics can be attributed to implicit learning per se, as researchers generally do not provide evidence to confirm whether implicit learning indeed occurred (eg, through self-report). For example, Welling *et al* 4 attributed positive effects of a video-instruction intervention on jump landing technique to implicit learning. However, participants almost exclusively reported to have focused *internally* on movement technique—suggesting *explicit learning* had occurred.

Moreover, the effects of a purely implicit or explicit ACL injury rehabilitation programme on actual secondary knee injury incidence rates have never been examined. Explicit verbal feedback on movement has, however, been identified as a key active ingredient in primary ACL injury risk reduction.^5^

It may be best to hedge our bets and avoid putting all our money on implicit learning strategies in the ACL rehabilitation context!

### Reappraising explicit motor learning

#### Tailoring to the individual

Individual differences in proprioception, working memory and motor learning preferences could influence the effectiveness of prescribing implicit or explicit learning interventions for ACL injury rehabilitation (see table 1)^6–9^:

**Table 1:** Individual constraints that could influence effectiveness of implicit and explicit motor learning interventions

	Individual factor		
	Proprioception	Verbal working memory	Athlete’s preference
Relevance in ACL rehabilitation	ACL serves role in proprioception High prevalence of proprioceptive impairment following ACL injury^S1^	Capacity differs from person to person High prevalence of ACL injury in young athletes^S2^, for whom working memory capacity is still developing^S3^	Preference for explicit/implicit learning differs from person to person People with musculoskeletal conditions strongly prefer to consciously control movements^S4.5^
Role in motor learning	Proprioceptive deficits compromise automatic movement control^S6,7^ Individuals with deficits benefit from explicit learning^S8–10^	Verbal working memory is key to process explicit movement instructions^S11^ Individuals with lower working memory capacity benefit from implicit learning^S11–13^	Individuals benefit from learning interventions that match their preference, for example, explicit strategies benefit those who prefer conscious control^S14.15^
Clinical recommendation	Consider more frequently using explicit learning interventions in case of proprioceptive deficits Screen proprioceptive deficits in both the affected and unaffected knee, using Joint-Position Sense tests^S1^	Consider more frequently using implicit learning interventions for athletes with poor verbal working memory Screen for verbal working memory deficits, for example, using Trail-Making Test B^S16^ or Automated Operation Span Test^S17^	Consider more frequently using motor learning method that aligns with athlete’s preference Screen preference for conscious control using Movement-Specific Reinvestment Scale^S18^

References contained in this table can be found in the ‘online supplemental files’.

10.1136/bjsports-2020-103643.supp1 Supplementary data

- Proprioceptive deficits are common following ACL injury.^10^ Proprioception is key to effective automatic movement control,^6^ and people with proprioceptive deficits benefit from explicit, rather than implicit, motor learning interventions.^7^
- Explicit learning is a cognitively demanding learning method. An individual needs to be able to process verbal instructions, keep these in mind and use these to guide movement execution. There is evidence that individuals with greater verbal working memory capacity benefit from explicit learning. Implicit learning seems more beneficial for individuals with *poorer* verbal working memory capacity.^8^
- Implicit or explicit learning appears most effective when it is aligned with an individual’s preference.^9^
  While not yet tested in the context of ACL rehabilitation, the above findings strongly warrant against an isolated, one-size-fits-all approach to motor learning. Screening these factors (table 1) may help guide decision-making for the prescription of implicit and explicit motor learning interventions during ACL injury rehabilitation.

### Explicit learning strategies to correct suboptimal movement patterns

Context in which athletes perform after ACL injury is also important. Explicit control is necessary for high-level, complex motor performance in cognitively demanding scenarios^11^ —those in which most non-contact ACL injuries occur. Explicit control allows the performer to intentionally correct inappropriate automatic motor responses, making small changes to technique as required.^11^ Elite athletes have shown to successfully use explicit interventions to de-automate, and subsequently improve, problematic movements.^11^ We therefore recommend caution against eradicating explicit motor learning interventions from ACL rehabilitation.

## Conclusion

Motor learning to regain sports-specific motor skills after ACL injury is not a ‘one-size-fits-all’ exercise. Coaches and healthcare practitioners need to blend implicit and explicit interventions, and tailor them according to personal and contextual factors. We argue that a blended approach has strong potential to improve outcomes of ACL injury rehabilitation.
