# Supplementary material for: Explicit motor learning interventions are still relevant for ACL injury rehabilitation: do not put all your eggs in the implicit basket!
Source: Br J Sports Med. 2021 Feb 15;56(2):63–4. doi: 10.1136/bjsports-2020-103643 (PMC8762022; doi:10.1136/bjsports-2020-103643)
Supplement: Supplementary data [file bjsports-2020-103643supp001.pdf]

SUPPLEMENTARY REFERENCES FOR TABLE 1

1. Relph N, Herrington L, Tyson S. The effects of ACL injury on knee proprioception: A meta-analysis. *Physiotherapy* 2014;100:187–95.
2. Sanders T, Maradit Kremers H, Bryan A et al. Incidence of anterior cruciate ligament tears and reconstruction: a 21-year population-based study. *Am J Sports Med* 2016;44:1502-7.
3. Spronk M, Vogel EK, Jonkman L. Electrophysiological evidence for immature processing capacity and filtering in visuospatial working memory in adolescents. *PLoS ONE.* 2012;7:e42262.
4. Street B, Adkin A, Gage W. Reported balance confidence and movement reinvestment of younger knee replacement patients are more like younger healthy individuals, than older patients. *Gait Posture* 2018;61:130-134.
5. Selfe J, Dey P, Richards J, et al. (2015). Do people who consciously attend to their movements have more self-reported knee pain? An exploratory cross-sectional study. *Clin Rehabil* 2015;29:95-100.
6. Clark D. Automaticity of walking: functional significance, mechanisms, measurement and rehabilitation strategies. *Front Hum Neurosci* 2015;9:246.
7. Henry M, Baudry S. Age-related changes in leg proprioception: Implications for postural control. *J Neurophysiol* 2019;112:525–38.
8. Ingram H, Van Donkelaar P, Cole J, et al. The role of proprioception and attention in a visuomotor adaptation task. *Exp Brain Res* 2000;132:114–26.
9. Kal E, Houdijk H, van der Kamp J, et al. Are the effects of internal focus instructions different from external focus instructions given during balance training in stroke patients? A double-blind randomized controlled trial. *Clin Rehabil* 2019;33:207–21.
10. Miall R, Kitchen N, Nam S, et al. Proprioceptive loss and the perception, control and learning of arm movements in humans: evidence from sensory neuronopathy. *Exp Brain Res* 2018;236:2137–55.
11. Buszard T, Farrow D, Verswijveren S, et al. Working memory capacity limits motor learning when implementing multiple instructions. *Front Psychol* 2017;8.
12. Kok M, Kal E, van Doodewaard C, et al. Tailoring instruction methods to the verbal working memory capacity of pupils with special needs can benefit learning outcomes in physical education. (in revision). Abstract published as: Effects of Explicit and Implicit Motor Instruction Methods in Pupils with Special Needs: The Relationship with Verbal and Visuospatial Working Memory. *J Sport Exerc Psychol* 2020;42:S44.
13. Van der Kamp, G., Kal, E., Kishna, K., et al. (in submission). Children's verbal and visuospatial working memory capacity influence effects of implicit and explicit motor learning on shot putting skill in physical education.
14. Tse A, van Ginneken W. Children's conscious control propensity moderates the role of attentional focus in motor skill acquisition. *Psychol Sport Exerc* 2017;31:35–9.
15. Maurer H, Munzert J. Influence of attentional focus on skilled motor performance: Performance decrement under unfamiliar focus conditions. *Hum Mov Sci 2013*;32:730–40.
16. Sánchez-Cubillo I, Periáñez J, Adrover-Roig D, et al. Construct validity of the Trail Making Test: role of task-switching, working memory, inhibition/interference control, and visuomotor abilities. *J Int Neuropsychol Soc* 2009;15:438-50.
17. Unsworth N, Heitz R, Schrock J, et al. An automated version of the operation span task. *Behav Res Method* 2005;37:498-505.
18. Masters R, Eves F, Maxwell J. Development of a movement specific reinvestment scale. Int Soc Sport Psychol (ISSP) World Congress 2005.
